# RACIAL DIFFERENCES IN THE ASSOCIATION BETWEEN SENSE OF CONTROL AND SOCIAL SUPPORT: A CROSS-LAGGED PANEL ANALYSIS

**DOI:** 10.1093/geroni/igad104.1224

**Published:** 2023-12-21

**Authors:** Eunbea Kim, Gina Lee, Jeong Eun Lee

**Affiliations:** Iowa State University, Ames, Iowa, United States; Iowa State University, Ames, Iowa, United States; Iowa State University, Ames, Iowa, United States

## Abstract

While it is widely acknowledged that having a sense of control and social support are crucial elements in promoting both physical and mental health among older adults, the relationship between these two factors and how they operate in diverse racial/ethnic settings is still unclear. To address this gap, we examined the bidirectional relationship between sense of control (constraints and mastery) and support received from three different resources (spouses, children, and friends) using cross-lagged panel regression analyses. Participants are White and Black Americans from four waves of Health and Retirement Study (N = 7,846 White; 944 Black). We found racial differences in the cross-over effects between social support and sense of control. In terms of constraints, while White Americans showed negative bidirectional associations between constraints and support from all resources across four waves, such associations were not observed in Black Americans. Additionally, positive bidirectional associations between mastery and support from all resources were found among White participants, whereas Black Americans showed only spill-over effects for mastery and support received from spouse and children across four waves. Notably, Black Americans’ support received from friends positively predicted their mastery across four waves. These findings underline the importance of considering racial/ethnic differences in understanding the complex interplay between social support and sense of control. Future studies should illuminate the causal relation and examine differential mechanisms in White and Black American older adults.

